# Cancers of the Appendix: Review of the Literatures

**DOI:** 10.5402/2011/728579

**Published:** 2011-08-11

**Authors:** Carl Ruoff, Louay Hanna, Wanqing Zhi, Ghulamullah Shahzad, Vladimir Gotlieb, Muhammad Wasif Saif

**Affiliations:** ^1^New York Hospital Medical Center of Queens, Flushing, NY 11355, USA; ^2^Nassau University Medical Center and North Shore LIJ Health System, East Meadow, NY 11554, USA; ^3^Division of Hematology/Oncology, College of Physicians and Surgeons, Columbia University, New York, NY, USA

## Abstract

Cancers of the appendix are rare. Most of them are found accidentally on appendectomies performed for appendicitis. When reviewed, majority of the tumors were carcinoid, adenoma, and lymphoma. Adenocarcinomas of appendix are only 0.08% of all cancers and the treatment remains controversial. Here we are reporting a 46-year-old male presented with symptoms of appendicitis, diagnosed with adenocarcinoma of the appendix. The patient was treated with appendectomy and refused further surgical intervention to complete hemicolectomy. Up to date, he remains asymptomatic. We performed literature review of the tumors of the appendix. Most of the benign conditions are treated with surgery alone. Lymphomas require CHOP-like chemotherapy and carcinoid syndrome treatment with somatostatin analogues. It is generally recommended that right hemicolectomy is the preferred treatment for adenocarcinoma of appendix. The role of chemotherapy is unclear due to lacking randomized trials but seems to be accepted if there is lymph node involvement or peritoneal seeding.

## 1. Introduction

The function of appendix in humans is unclear, although it is thought to possibly play a role in the immune system. Very rarely, the appendix may become cancerous. Cancer of the appendix may cause appendicitis or rupture of appendicitis. Mostly this is the first symptom of appendix cancer.

The majority of appendiceal tumors are carcinoids, while the remaining 10% to 20% are mucinous cyst-adenocarcinoma, adenocarcinoma, lymphosarcoma, paraganglioma, and granular-cell tumors. Most common symptoms include acute pain in right lower quadrant and other symptoms of inflammation like fever, leukocytosis, and so forth. Appendectomy is performed as clinically indicated. If a mass in the appendix is encountered incidentally during the course of abdominal surgery, an appendectomy is performed with frozen-section analysis of the mass. Most masses are benign mucoceles or very small carcinoids. They do not require any further management. However, if lymphoma or larger carcinoid was identified, chemotherapy or more extensive surgery will be required. When the mass is adenocarcinoma, the treatment algorithm is less defined as the data remain controversial.

## 2. Case Report

We are reporting a 46-year-old African-American male presented to the emergency room with lower back pain secondary to a motor vehicle accident in 2009. After admission, patient complained of right lower quadrant pain, low-grade fever, and nausea. Laboratory showed leukocytosis. Patient was taken to the operating room and underwent appendectomy. During the surgery, the appendix was found to be gangrenous and perforated with an associated abscess. The surgical pathology showed adenocarcinoma of appendix, moderately differentiated with no lymphovascular invasion, and acute appendicitis. The tumor was found to be 2.3 cm, grade 2, mucin-producing adenocarcinoma. Postoperative course was uneventful. The patient returned to work after two weeks. Subsequently, he came to oncology clinic for further recommendations. The patient underwent staging CT scans of the chest, abdomen, and pelvis with contrast which did not reveal any evidence of residual malignancy. Because of the size of the tumor and the grade, we offered him to have right hemicolectomy. The patient was evaluated by a colorectal surgeon and subsequently refused the procedure. He also declined consideration of adjuvant chemotherapy. After 3 months of followup, he remained asymptomatic and did not keep his appointments after that.

## 3. Literatures Review

### 3.1. Epidemiology and Classification

Cancer of the appendix is very rare and is typically found incidentally in approximately 1% of appendectomies [1]. According to a report published by the National Cancer Institute using the Surveillance, Epidemiology, and End Results (SEER) database, appendiceal neoplasms account for approximately 0.4% of gastrointestinal tumors [2].

Even though primary appendiceal cancers are rare, there is a diverse histology. Carcinoids are by far the most common, accounting for approximately 66%, with cyst-adenocarcinoma accounting for 20% and adenocarcinoma accounting for 10% [1]. Then there are the rare forms of cancers which include adenocarcinoid, signet ring, non-Hodgkin's lymphoma, ganglioneuroma, and pheochromocytoma. Benign primary processes are mainly mucinous epithelial neoplasms, also called adenomas, cystadenoma, and benign neoplastic mucocele (Table 1). Frequency of appendiceal neoplasms found in 7970 appendectomy specimens is shown in Table 1 [2].

The majority of primary cancers of the appendix occur in 55–65 years of age, except for malignant carcinoid, which has a mean age diagnosis of 38. Men and women seem to be at equal risk for all appendiceal neoplasms except for malignant carcinoid which may have woman to man ratio in excess of 3 : 1, depending on the studies that are analyzed [2].

### 3.2. Carcinoid Tumors

In 1907, German pathologist Dr. Oberndorfer described a collection of tumors he found on the small intestine as looking like carcinoma but also exhibiting features of benign adenomas, as carcinoid (carcinoma-like) [3]. In 1914, Dr Gosset and Dr Mason postulated that carcinoid tumors were made up of enterochromaffin cells, a type of neuroendocrine cells within the lamina propria and submucosa. These cells produce and contain approximately 90% of the serotonin in our bodies [4]. 

It has been widely accepted that the majority of carcinoids arise in the appendix,; however, recent data suggests that other locations, such as the rectum and small intestine may actually be more common [5]. In a study by Mggard et al., 11,427 cases were analyzed, and it was found that 44.7% of tumors were found in the small intestine, 19.6% in the rectum, 16.7% in the appendix, 10.6% in the colon, and 7.2% in the stomach [5]. Data from the SEER database also suggest that there is a greater proportion of pulmonary and gastric carcinoids compared to appendiceal carcinoids, however, these changes may be due to variation in reporting as benign looking carcinoids were not added to the SEER database until 1986 [6].

The incidence and prevalence of carcinoid tumors have conflicting statistics. In many of the older articles, incidence was stated as one to two in 100,000 [7, 8]. Newer data show that the incidence may be double that. The inconsistency is due in part to the fact that the disease is mostly asymptomatic and can remain asymptomatic for many years, so the incidence rate is more likely to be higher. Some research has stated that carcinoid tumors are found in 1% of necropsies. This would give a prevalence rate much higher than was previously accepted.

Most carcinoid tumors of the appendix are asymptomatic. The average time for a carcinoid tumor to become symptomatic is 9 years [9]. When the tumor is located in the tip of the appendix, which it is in approximately 75% of the cases, it generally does not present with symptoms until it becomes metastatic. When the tumor is located at the base of the appendix, it can occlude the lumen and give the patient similar signs and symptoms of appendicitis [1]. In these patients, the diagnosis of carcinoid cancer is typically made by pathology after an appendectomy has been performed.

In rare cases, the patient can present with signs and symptoms related to a carcinoid syndrome. These include flushing, tachycardia, severe explosive diarrhea, and hypotension. These effects are mostly caused by the serotonin that the enterochromaffin cells are producing. The carcinoid tumor also produces vasoactive substances such as histamine, prostaglandins, kallikrein, bradykinins, substance P, gastrin, corticotrophin, and neuron-specific enolase. The lungs and the liver are able to clear many of these agents along with the serotonin, therefore, able to avoid carcinoid syndrome. It is not until these organs have carcinoid metastasis that the ability to clear these substances becomes impaired, and symptoms of carcinoid syndrome become apparent [9]. Carcinoid syndrome affects approximately 10% of those with carcinoid tumors.

As stated before, diagnosis of carcinoid tumor of the appendix is usually made after an appendectomy. When the patient presents with signs and symptoms of appendicitis, a CAT scan of the abdomen is typically performed. These imaging studies usually show a process of acute appendicitis or have associated calcifications [10, 11]. 

In a patient with suspected carcinoid syndrome, measuring the urinary excretion of 5-HIAA and serum chromogranin A levels can be useful in diagnosis [9]. An OctreoScan may be beneficial; however, its results are not always reliable. 

The extent of surgery is based upon the size of the tumor, but since the majority of carcinoid tumors are found incidentally on simple appendectomies, a second surgery is sometimes needed. The National Comprehensive Cancer Network (NCCN) guidelines for treatment of carcinoid tumors state that tumors <2 cm confined to the appendix can be treated with simple appendectomy with no followup required. For tumors >2 cm, or those with extra-appendigeal invasion, an appendectomy with right hemicolectomy and cytoreductive surgery is necessary. Post-operatively, a 3 month followup which includes a history and physical (H&P), CT of the abdomen, and tests for markers (5-HIAA and chromogranin A) should be completed [2]. 

For patients with metastatic disease, somatostatin analogs can be beneficial in relieving the symptoms of carcinoid syndrome. Somatostatin, an 18 amino-acid peptide, binds to somatostatin receptors to block the secretion of hormones such as growth hormones, gastrin, insulin, and glucagon. These receptors are found on over 80% of carcinoid tumors [12]. Octreotide, an eight-amino-acid, long-acting somatostatin analogue, works through G-protein activation on somatostatin receptor subtypes 2, 3, and 5 [13]. octreotide was effective in decreasing symptoms in 88% of patients and decreasing the urinary 5-HIAA in 72% of patients [6]. In patients who do not respond to octreotide, interferon-alpha has been added with some positive results; however, as stated by Mayer, this therapy comes at a cost of side effects which may include fever, fatigue, anorexia, and weight loss [6].

Generally, the prognosis for carcinoid tumors of the appendix is very good. If the tumor is confined to the appendix, the disease is said to have 94 percent 5-year survival rate. For patients with regional disease, there is an 85 percent 5-year survival rate, and for distant metastasis, which occurs in approximately 4% of the time in appendiceal carcinoid-tumors, there is a 34 percent 5 year survival rate [8]. There is still no accepted TMN-based staging system for carcinoid.

### 3.3. Benign Appendix Tumors

The endothelium of the vermiform appendix is composed of masses of lymph tissue and is lined by mucous secreting columnar cells. Changes to the columnar cells layer cause the four different types of pathology listed below.

1. Retention cyst.
2. Villous Hyperplasia.
3. Cystadenoma.
4. Cystadenocarcinoma.

Many consider these changes to be a spectrum of disease much like colonic polyp formation and progression to carcinoma in colon cancer. Like colon cancer, there are both hyperplastic and adenomatous changes at the cellular level [12].

All of these changes cause the formation of a mucocele, which is defined as a dilation of the appendix due to excessive mucinous production. The mucin builds up due to a malignant or non malignant obstruction of the outflow tract.

**Retention Cysts**

They occur when there is a nonmalignant obstruction of the outflow tract of the appendix. The most common cause of the obstruction is a fecalith. The epithelial cells continue to produce mucin, and the appendix becomes distended. There are no changes at the cellular level.

The vast majority of mucoceles measure less than 2 cm, and those that exceed 2 cm are more likely to be neoplasms [10, 11]. Most are clinically asymptomatic and are found incidentally as palpable masses in the lower right quadrant or on imaging performed for other reasons. On plain abdominal films, mucoceles are difficult to appreciate. CT and MRI are more effective in identifying the extent of the mass. Treatment is primarily surgical excision.

**Villous Hyperplasia**

It is also called hyperplastic polyp, and has many of the same qualities of colonic polyps. The polyps are sessile or flat and do not have well-defined borders. The involvement is isolated to the mucosa. Instead of a fecalith causing the obstruction as seen in retention cysts, the obstruction is caused by the hyperplastic polyp. Presentation, imaging, and treatment are similar to the retention cyst.

**Cystadenomas**

They are the most common neoplasm of the appendix. Much like the villous hyperplastic polyp, the growth itself is what causes the formation of a mucocele. Histologically, the lesion is benign; however, there may be a continuum to cystadenocarcinomas. Most present as acute appendicitis, and the treatment is surgical excision. Rupture of the appendix can cause a phenomenon called pseudomyxoma peritonei which will be discussed later.

### 3.4. Malignant Appendix Tumors

**Adenocarcinoma**

Of the appendix is very rare, accounting for 0.5% of all gastrointestinal cancers [14]. Within the adenocarcinoma malignancies there are three subtypes: mucinous (55%), colonic type (34%), and adenocarcinoid (11%) which has a mixed morphology. The mean age of diagnosis is in the fifth decade of life, with an even male to female ratio for all but colonic type, which may have a higher incidence in men [1]. The incidence of adenocarcinoma has been stated to be from 0.004% to 0.08% [15].

**Mucinous Adenocarcinoma**

It is the malignant counterpart to the mucinous adenoma. Both present with similar symptoms. Adenocarcinoid, also called Goblet cell carcinoid, has features of both carcinoid tumor and mucinous adenocarcinoma. They account for 5% of cancers of the appendix, with an average age diagnosis of 58 years, and an even distribution between men and women [1].

As is the case with most cancers of the appendix, the most common presentation is that of acute appendicitis. This may be from distention of the appendix causing pain or from a superinfection. Those with mucinous adenocarcinoma may present with a gradually distending abdomen which may be seen with pseudomyxoma peritonei. 

The mucinous type, also called mucinous cyst-adenocarcinoma, causes a mucocele by the neoplasm occluding the narrow lumen which allows the mucin to build up and distend the appendix. Perforation may occur allowing the spill of cancerous cells out into the peritoneum, which creates the condition of pseudomyxoma peritonei. These cells then seed the organs of the peritoneum and continue to produce the mucin. As the mucin accumulates, the abdomen becomes distended which is referred to as “jelly belly” [16]. Colonic type adenocarcinoma is less likely to present with a mucocele. Instead, they are more likely to present with a focal mass in the right lower quadrant.

**Adenocarcinoma**

Adenocarcinoma of the appendix is rarely diagnosed preoperatively. In a study by Nitecki et al., none of the 96 patients with adenocarcinoma of the appendix were diagnosed preoperatively, and it was only considered in the differential diagnosis in 10 patients.

CT imaging is helpful in identifying a mucocele caused by the neoplasm. Calcifications increase the likelihood that this process is malignant and not infective or inflammatory. If there is a superinfection associated with the malignancy, there may be air bubbles present on the CT. If the patient presents with a distended abdomen due to pseudomyxoma peritonei, a CT would show widespread heterogeneous locules in the peritoneal cavity [10, 11]. Since there are no imaging studies specific for diagnosing adenocarcinomas, the final diagnosis is most often made postoperatively on microscopic examination. 

It has been generally accepted that a right hemicolectomy is the preferred surgical intervention for all subtypes of adenocarcinoma. While some surgeons suggest that a simple appendectomy is sufficient for tumors exhibiting only local disease, many studies have shown that there is a clear survival benefit to the addition of a hemicolectomy [14, 17, 18]. In a study by Nitecki, the 5-year survival rate for hemicolectomy was 73% versus 44% in the appendectomy group. These studies have found that the colonic and goblet cell subtypes are invasive, and approximately half the patients present with nodal metastasis [14]. However, there are some studies that disagree. Gonzalez-Moreno and Sugarbaker found that those patients with mucinous type cancer had no survival benefit from hemicolectomy versus appendectomy [19]. They further mention that hemicolectomy is recommended in those patients where (1) it is necessary to clear the tumor or perform complete cytoreduction; (2) lymph node involvement is demonstrated by histopathological examination of the appendiceal or ileocolic lymph nodes; or (3) a nonmucinous subtype is identified by histopathological examination.

 In a study done by Pahlavan and Kanthanon adenocarcinoid tumors, he states that even though Goblet cell carcinoma is an aggressive tumor, a simple appendectomy is appropriate in most cases. However, he further states that a right hemicolectomy should be performed in the following scenarios: (1) cellular undifferentiation, (2) increased mitotic activity, (3) involvement of the base of the appendix, (4) lymph node metastasis, or (5) tumor size greater than 2 cm [20]. These guidelines allow the surgeon some direction in deciding whether or not to reoperate after a patient has had an appendectomy for an apparent appendicitis.

While there are small studies and anecdotal case reports that suggest a response to regimens containing 5-FU, the role of chemotherapy has yet to be clearly defined. Most oncologists agree that in the presence of nodal involvement, systemic and intraperitoneal chemotherapy regimens should be used [14, 16, 20]. Sugarland suggests postoperative intraperitoneal chemotherapy in the setting of pseudomyxoma peritonei, as long as cytoreduction and debulking have been accomplished, reducing recurrence rates [16].

Prognosis of adenocarcinoma depends on the subtype and extent of disease. Mucinous adenocarcinoma is considered to have a more favorable prognosis because it does exhibit hematogenous or lymphatic spread [16, 17]. While it would be natural to assume that those patients with intraperitoneal seeding due to appendiceal perforation, would have a worse prognosis versus those who did not. However, in a study by Nitecki and others, there actually was no difference in 5-year survival rates between the two groups [14]. Perforation did in often times lead to earlier medical intervention and treatment.

Goblet cell subtype is considered to have a worse prognosis with one study listing a 55% 5-year survival rate while Pahlavan states a 60–80% 5-year survival rate [17, 20]. The discrepancy may be due to differences in lymph node involvement for the two studies. The first had 43% lymph node involvement where Pahlavan only showed an 8.76% involvement.

**Lymphoma**

The gastrointestinal tract is the most common site for extranodal lymphoma. The stomach is the most common, followed by the small intestine, pharynx, colon, and esophagus. Lymphoma of the appendix is almost exclusively non-Hodgkin's B-cell lymphoma, more specifically, Burkitt's lymphoma. The incidence of primary appendiceal lymphoma has been estimatedat 0.015% of appendectomy specimens [21]. Men are more likely to develop appendiceal lymphoma over women by 1.5 : 1, with a median age onset of 18 years [22].

As so many other primary tumors of the appendix, patients typically present with symptoms similar to acute appendicitis. Patients may also present with a more insidious onset of pain in the right iliac fossa for a few months and an associated palpable mass in the right lower quadrant.

When imaging is done, the appendix demonstrates prominent enlargement, while it maintains the vermiform appearance [10, 11]. On ultrasound, the diffuse thickening is hypoechoic and often mimics the cystic dilation of the lumen seen in mucoceles. 

As with all lymphoma's, chemotherapy is the mainstay of treatment. The classic combination of cyclophosphamide, doxorubicin, vincristine, and prednisone (CHOP) has been used for many decades, and since the Groupe d'Etude des Lymphomas de l'Adulte study, Rituximab has been added [23]. Rituximab, a monoclonal antibody against CD20, was found to increase complete response rates from 64% to 76% [24]. It was also found that the CHOP plus Rituximab increased event free states and overall survival. The combination of CHOP and rituximab is now the standard of care for large B-cell lymphoma in the US. For Burkitt's lymphoma, a more aggressive chemotherapy regimen is in need.

**Primary Signet Ring Cell Carcinoma**

Primary Signet Ring Cell Carcinoma of the appendix is a very rare cancer. According to the data gathered from the SEER database, the incidence is approximately 0.15, with an average age of 59, and there is no male or female preference. Of all the subtypes of appendiceal cancers, signet ring had the lowest percentage of localized disease at 14%, and the highest percentage of distant disease at 60%. The most common presentation is of acute appendicitis, and the recommended surgical intervention is of a right hemicolectomy [25]. The prognosis is considered very poor [1]. Of all the subtypes, signet ring had the lowest overall 5-year survival rate at 18%. Those who presented with distant disease had a 5-year survival of 7%. The role of chemotherapy as a treatment in signet ring cancers of the appendix has not been determined.

**Ganglioneuroma**

It is an extremely rare cancer that is associated with neurofibromatosis, MEN 2b, congenital defects, carcinomas, and various polyp forming diseases [13]. A search of the literature showed only three case reports of ganglioneuromas involving the appendix, all being the Neurofibromatosis type 1 [26–28]. Two of the cases presented as discomfort in the lower right quadrant, while the third presented as a palpable mass found on physical exam. All three patients underwent surgical removal of the tumor and the involved surrounding tissue.

In those patients with Neurofibromatosis 1 and MEN 2b, pheochromocytomas are also prevalent and may be found as a primary cancer of the appendix. This is extremely rare, and there is very little data available to extrapolate meaningful conclusions at this time.

## 4. Discussion

Appendix cancer is rare, and most commonly found incidentally in an appendectomy specimen that was obtained for an unrelated condition. The main histologic types are carcinoids, adenocarcinomas, adenocarcinoids, cystadenomas, and cystadenocarcinomas. For appendiceal carcinoids, reoperation and right colectomy is recommended for tumors larger than 2 cm and for smaller tumors with mesoappendiceal invasion. Most patients have localized disease, and the prognosis is excellent. 

The spectrum of epithelial tumors of the appendix ranges from the benign mucocele to an aggressive adenocarcinoma. Simple appendectomy, taking care not to rupture the tumor intraoperatively, is sufficient therapy for benign appendiceal mucoceles, cystadenomas, and some cystadenocarcinomas. A right colectomy is indicated for cystadenocarcinomas with mesenteric or adjacent organ involvement and complicated mucoceles with involvement of the terminal ileum or cecum, cystadenocarcinomas. 

Pseudomyxoma peritonei (PMP) is a unique condition characterized by diffuse collections of gelatinous material in the abdomen and pelvis, associated with mucinous implants on the peritoneal surfaces. The term should be reserved for the clinical situation in which a ruptured cystadenoma seeds the peritoneal cavity with mucus-producing epithelial cells, termed diffuse peritoneal adenomucinosis (DPAM). The natural history is one of indolent but progressive growth, and if left untreated, this is a fatal condition. Standard treatment for PMP is repeated surgical debulking for symptomatic disease. This treatment is not curative but aims to limit the buildup of mucus and its pressure effect. A more aggressive approach using radical surgical cytoreduction of all intraabdominal and pelvic disease and intraperitoneal heated chemotherapy (IPHC) has been adopted by some clinicians, aiming for cure. Although randomized trials have not been conducted, five-year survival rates of 70 to 86 percent have been reported for highly selected patients. The importance of surgical technique and surgeon experience to the success and safety of this approach cannot be overemphasized.

In contrast to other appendiceal tumors, adenocarcinomas more often present with a clinical picture of acute appendicitis. Standard treatment is a right colectomy. The role of adjuvant chemotherapy for adenocarcinoma of the appendix is unknown. Despite the lack of available data, many oncologists recommend adjuvant 5-FU-based chemotherapy particularly for patients with node-positive intestinal type adenocarcinoma, extrapolating from data on adjuvant chemotherapy for node-positive colon cancer. Optimal treatment of patients with intraperitoneal dissemination of appendiceal adenocarcinoma (mucinous peritoneal carcinomatosis as distinguished from DPAM) is unclear. Selected patients treated with aggressive surgical cytoreduction and IPHC may do well-long term, but patient selection and the experience of the treating team are critical. In uncontrolled series from experienced institutions, long-term survival rates in highly selected patients range from 28 to 72 percent at three to 10 years. The benefit of systemic chemotherapy for advanced disease is unknown. Although mucinous appendiceal adenocarcinomas have been thought to be relatively chemotherapy refractory, case reports suggest some level of benefit for therapy in individual patients. 

The cancers of the appendix could represent a challenge for diagnosis and management. No standard of care is established due to rare frequency of occurrence, our case and the literature review are a summary of the appendix tumor, and the management based more on personal opinion of the oncologists who deal with this rare type of tumor, hopefully, would help in the future to create clinical trials to establish more evidence-based medicine help in the management.

## Figures and Tables

**Table 1:** Incidence of appendiceal neoplasms found in 7970 appendectomy specimens.

Appendiceal neoplasm	*N*
Carcinoid	42
Benign tumors	
Mucinous cystadenoma	7
Villous adenoma	5
Malignant tumors	
Adenocarcinoma	8
Lymphoma	1
Metastasis	11

Total	74

## References

[1] McCusker ME, Coté TR, Clegg LX, Sobin LH (2002). Primary malignant neoplasms of the appendix: a population-based study from the surveillance, epidemiology and end-results program, 1973–1998. *Cancer*.

[2] Connor SJ, Hanna GB, Frizelle FA (1998). Retrospective clinicopathologic analysis of appendiceal tumors from 7,970 appendectomies. *Diseases of the Colon and Rectum*.

[3] Läuffer JM, Zhang T, Modlin IM (1999). Current status of gastrointestinal carcinoids. *Alimentary Pharmacology and Therapeutics*.

[4] Gosset A, Masson P (1914). Tumeurs endocinne de l'appendice. *La Presse Medicale*.

[5] Maggard MA, O’Connell JB, Ko CY (2004). Updated population-based review of carcinoid tumors. *Annals of Surgery*.

[6] Kulke MH, Mayer RJ (1999). Carcinoid tumors. *New England Journal of Medicine*.

[7] Godwin JD (1975). Carcinoid tumors. An analysis of 2837 cases. *Cancer*.

[8] Modlin IM, Sandor A (1997). An analysis of 8305 cases of carcinoid tumors. *Cancer*.

[9] Robertson RG, Geiger WJ, Davis NB (2006). Carcinoid tumors. *American Family Physician*.

[10] Pickhardt PJ, Levy AD, Rohrmann CA, Kende AI (2003). Primary neoplasms of the appendix: radiologic spectrum of disease with pathologic correlation. *Radiographics*.

[11] Pickhardt PJ, Levy AD, Rohrmann CA, Kende AI (2003). Erratum: Primary neoplasms of the appendix: radiologic spectrum of disease with pathologic correlation. *Radiographics*.

[12] Cappell MS (2005). The pathophysiology, clinical presentation, and diagnosis of colon cancer and adenomatous polyps. *Medical Clinics of North America*.

[13] Patel YC, Srikant CB (1994). Subtype selectivity of peptide analogs for all five cloned human somatostatin receptors (hsstr 1–5). *Endocrinology*.

[14] Nilecki SS, Wolff BG, Schlinkert R, Sarr MG (1994). The natural history of surgically treated primary adenocarcinoma of the appendix. *Annals of Surgery*.

[16] Sugarbaker PH, Kern K, Lack E (1987). Malignant pseudomyxoma peritonei of colonic origin. Natural history and presentation of a curative approach to treatment. *Diseases of the Colon and Rectum*.

[17] Hartley JE, Drew PJ, Qureshi A, MacDonald A, Monson JRT (1996). Primary adenocarcinoma of the appendix. *Journal of the Royal Society of Medicine*.

[18] Ferro M, Anthony PP (1985). Adenocarcinoma of the appendix. *Diseases of the Colon and Rectum*.

[19] González-Moreno S, Sugarbaker PH (2004). Right hemicolectomy does not confer a survival advantage in patients with mucinous carcinoma of the appendix and peritoneal seeding. *British Journal of Surgery*.

[20] Pahlavan PS, Kanthan R (2005). Goblet cell carcinoid of the appendix. *World Journal of Surgical Oncology*.

[21] Collins DC (1963). 71,000 human Appendix specimens. A final report summerising 40 years' study. *American Journal of Proctology*.

[22] Stewart RJ, Mirakhur M (1986). Primary malignant lymphoma of the appendix. *Ulster Medical Journal*.

[23] Cheson BD, Leonard JP (2008). Monoclonal antibody therapy for B-cell non-Hodgkin’s lymphoma. *New England Journal of Medicine*.

[24] Coiffier B, Lepage E, Brière J (2002). Chop chemotherapy plus rituximab compared with chop alone in elderly patients with diffuse large-B-cell lymphoma. *New England Journal of Medicine*.

[25] McGory ML, Maggard MA, Kang H, O’Connell JB, Ko CY (2005). Malignancies of the appendix: beyond case series reports. *Diseases of the Colon and Rectum*.

[26] Merck C, Kindblom LG (1975). Neurofibromatosis of the appendix in von Recklinhausen’s disease: a report of a case. *Acta Pathologica Microbiologica Scandinavica A*.

[27] Lie KA, Lindboe CF, Kolmannskog SK, Haugen SE, Grammeltvedt AT (1992). Giant appendix with diffuse ganglioneuromatosis. An unusual presentation of von Recklinghausen’s disease. Case report. *European Journal of Surgery*.

[28] Lockhart ME, Smith JK, Canon CL, Morgan DE, Heslin MJ (2000). Appendiceal ganglioneuromas and pheochromocytoma in neurofibromatosis type I. *American Journal of Roentgenology*.

